# Changes in Oral Health-Related Quality of Life Following Restorative Dental Treatment of Untreated Dental Caries: A Prospective Study in the Saudi Population

**DOI:** 10.3290/j.ohpd.c_2758

**Published:** 2026-07-06

**Authors:** Azhar Iqbal, Farooq Ahmad Chaudhary, Saeed Alqahtani, Hmoud Ali Algarni, Meshal Aber Alonazi, Yasir Alyahya, Bilal Arjumand, Yasir Dilshad Siddiqui, Kumar Chandan Srivastava

**Affiliations:** a Azhar Iqbal Associate Professor, Department of Restorative Dentistry, College of Dentistry, Jouf University, Sakaka, Saudi Arabia. Conceptualization, statistical analysis, wrote the manuscript.; b Farooq Ahmad Chaudhary Associate Professor, School of Dentistry, Shaheed Zulfiqar Ali Bhutto Medical University, Islamabad, Pakistan. Conceptualization, supervision, data analysis, wrote and proofread the manuscript.; c Saeed Alqahtani Assistant Professor, Department of Restorative Dentistry, College of Dentistry, Jouf University, Sakaka, Saudi Arabia. Data collection and analysis, contributed substantially to discussion, proofread the manuscript.; d Hmoud Ali Algarni Associate Professor, Department of Restorative Dentistry, College of Dentistry, Jouf University, Sakaka, Saudi Arabia. Data collection, proofread the manuscript.; e Meshal Aber Alonazi Associate Professor, Department of Restorative Dentistry, College of Dentistry, Jouf University, Sakaka, Saudi Arabia. Data collection, statistical analysis, proofread the manuscript.; f Yasir Alyahya Assistant Professor, Department of Conservative Dental Sciences, College of Dentistry, Qassim University, Buraydah, Qassim, Saudi Arabia. Statistical analysis, wrote the results section, proofread the manuscript.; g Bilal Arjumand Associate Professor, Department of Conservative Dental Sciences, College of Dentistry, Qassim University, Buraydah, Qassim, Saudi Arabia. Contributed substantially to data collection and discussion, proofread the manuscript.; h Yasir Dilshad Siddiqui Assistant Professor,Department of Preventive Dentistry, College of Dentistry, Jouf University, Sakaka, Saudi Arabia. Contributed substantially to data collection and analysis, discussion, proofread the manuscript.; i Kumar Chandan Srivastava Associate Professor, Department of Oral Maxillofacial Surgery and Diagnostic Sciences, College of Dentistry, Jouf University, Sakaka, Saudi Arabia. Supervisor, advisor, performed statistical analysis, proofread the manuscript.

**Keywords:** dental caries, dental restoration, general health, oral health, quality of life.

## Abstract

**Purpose:**

The effect of restorative dental treatment on oral health-related quality of life (OHRQoL) and general well-being in Saudi adults is not well-documented. This study therefore evaluated the sensitivity of the OHIP-14, OIDP, and WHOQOL-BREF instruments in capturing changes in OHRQoL and health-related quality of life (HRQoL) after restorative care for caries.

**Materials and Methods:**

Between September 2024 and March 2025, a prospective longitudinal cohort of 269 adult patients with untreated caries was recruited from Jouf University Dental Clinics in Saudi Arabia. Standardized restorative protocols were followed for all treatments. The three validated Arabic version of OHIP-14, OIDP, and WHOQOL-BREF instruments were used to assess the quality of life at baseline and at a one-month follow-up. To examine discriminant validity, responsiveness of the instruments, and factors predicting therapeutic change, statistical analysis involved paired and independent t-tests, effect size calculation (Cohen’s d), and multiple linear regression.

**Results:**

Statistically significant post-treatment improvements were observed across all subdomians of the three instruments. Mean OHIP-14 and OIDP scores decreased markedly (20.64 to 6.81 and 7.17 to 0.27, respectively; p < 0.001), while WHOQOL-BREF scores increased statistically significantly (32.53 to 43.03; p < 0.001). The degree of improvement was linked to initial caries severity. Therefore, the participants having more advanced caries at the start of the study demonstrated greater responsiveness, particularly in their scores on the OHIP-14 and WHOQOL-BREF.

**Conclusion:**

The restorative dental treatment of dental caries statistically significantly improves the oral health-related and general quality of life of adult patients. The responsiveness of the OHIP-14, OIDP, and WHOQOL-BREF instruments, as demonstrated by the current study findings, supports their application as robust patient-reported outcome measures (PROMs) in clinical dentistry. It is recommended that these PROMs should be integrated into practice to advance a more holistic and patient-centered approach to the provision of oral healthcare.

Dental caries is one of the most common globally prevalent problems affecting both oral and general health.^24^ The permanent teeth of around 2.3 billion adults are affected by caries, as are over 530 million children worldwide, thus making it one of the most common chronic infectious diseases in humans.^13^

It is a multifactorial disease influenced by microbiological, immunological, genetic, environmental, and behavioral factors, all of which determine its severity.^15^ Although it is largely preventable, in advanced stages it may lead to tooth sensitivity, pain, infection, and ultimately tooth loss, hence negatively affecting function, speech, and appearance.^33^ Therefore, the quality of life related to oral health and overall well-being can be significantly impaired by these adverse outcomes of untreated caries.^16^

In the past twenty years, due to a paradigm shift towards patient-centered care, healthcare outcomes have been redefined, thus positioning patient satisfaction and self-perceived quality of life as central indicators of success.^16^ The quality of life is inherently multidimensional, involving an intricate interplay among social, psychological, and physical health, including oral health status.^6^ Consequently, the “total patient care” concept has emerged, thus emphasizing a holistic approach that manages the disease itself alongside its wider psychosocial and functional consequences. This evolution of total patient care highlights a critical gap and opportunity for modern dentistry, i.e., how the restorative treatment of caries translates into measurable improvements in patients’ overall quality of life, thereby aligning clinical objectives with comprehensive patient well-being. Multiple validated instruments have been developed to measure these complex dimensions of quality of life.^9,25^ An individual’s oral-health–related quality of life (OHRQoL) is closely linked to the presence and consequences of caries.^16^ More precisely, OHRQoL is directly related to a patient’s number of decayed and missing teeth. Dental treatment significantly affects the patient’s overall quality of life (QoL). A variety of instruments measuring OHRQoL are used to assess outcomes of those treatments.^25^

The Oral Health Impact Profile-14 (OHIP-14) and the Oral Impacts on Daily Performances (OIDP) index are the two most established tools.^1,11,28^ Both tools have been validated for their strong psychometric properties, including high validity and internal consistency for detecting changes in quality of life following restorative treatment.^5,29^ The OHIP-14 has been specifically recognized as a reliable and sensitive tool of OHRQoL, capable of detecting differences before and after intervention across various oral health conditions.^28^ The OIDP, being a well-established and validated instrument, also measures the functional and psychosocial effects of oral health on the everyday life of individuals. It has been used in many different populations.^1^ The World Health Organization’s Quality of Life – Brief Version (WHOQOL-BREF) is the most commonly used instrument measuring the overall health-related quality of life (HRQoL).^31^ An association between the prevalence of caries and reduced QoL of adult populations has been demonstrated by a large body of evidence.^6,16^ Specifically, this association is supported by studies showing a negative correlation between the number of remaining teeth and the OHRQoL. Furthermore, those individuals having poor OHRQoL scores are likely to have more untreated carious teeth.^2,14^

Although previous studies demonstrated associations between caries and QoL, only a limited number of studies exist which evaluate whether or to what degree restorative dental treatments can improve OHRQoL and HRQoL.^32^ Therefore, the present study aimed to bridge this important gap in the literature by assessing responsiveness of three quality of life instruments (OHIP-14, OIDP, and WHOQOL-BREF) to detect the differences in OHRQoL and HRQoL among Saudi adults before and after receiving restorative dental treatment. The findings were intended to assess the utility of these tools in clinical practice and to support an evidence-based, more holistic patient-centered approach to oral healthcare within this specific context.

## MATERIALS AND METHODS

This prospective longitudinal quantitative study was conducted between September 2024 and March 2025 at the educational dental clinic center of the College of Dentistry, Jouf University, Saudi Arabia. The study aimed to evaluate three OHRQoL instruments – the Oral Health Impact Profile-14 (OHIP-14), the Oral Impacts on Daily Performances (OIDP), and the World Health Organization Quality of Life assessment (WHOQOL-BREF) – for their responsiveness to the restorative treatment of caries. This study adopted a pragmatic research paradigm with a dominant quantitative (positivist) orientation^23^ to test the responsiveness of these instruments within real-world clinical settings. In other words, emphasis was placed on the practical applicability and utility of OHRQoL measures in real clinical dental context. The participants presenting with untreated caries were evaluated at baseline (prior to treatment) and subsequently re-evaluated one month following the completion of dental treatment.

A-priori sample size calculations for this research were completed using G*Power software (Version 3.1.9.4). The required sample size for the paired t-test was determined to be a minimum of 269 participants with an alpha of 0.05, a power of 0.9, and an anticipated small effect size (Cohen’s d = 0.2). However, to accommodate an estimated 5% drop-out rate, the final target sample size was adjusted to 279. Therefore, a total of 279 participants were recruited at baseline from the educational dental clinic center of the College of Dentistry, Jouf University, Saudi Arabia. A stratified consecutive non-probability sampling technique was used to recruit the participants for current study. The participants were sequentially enrolled until the target sample was achieved. The rationale behind choosing this sampling technique was to ensure the practicality in a clinical setting and to capture a sample representative of patients seeking care for dental caries during the defined study period. However, as a non-probability sample drawn from a university dental clinic, the study population may not fully represent the general adult Saudi population.

Eligibility criteria included adults (≥18 years) presenting with active dental caries necessitating restorative treatment, who were fluent Arabic speakers and provided consent for follow-up.

However, the participants with systemic conditions affecting oral health, current orthodontic treatment, root caries, post-restorative complications (pulpitis or necrosis), previous root canal treatment, or any cognitive impairment precluding questionnaire completion were excluded from the current study. The current study followed the guidelines of the Declaration of Helsinki and ethical approval was provided by the Local Committee of Bioethics at the College of Dentistry, Jouf University under Bioethical Approval Number (Ref. no. 1-09-45). Participants were made aware of the purpose of the study and were also informed that their involvement in the present study was voluntary.

No monetary or other type of incentive was given for participation in the survey. All participants’ information was anonymous to protect their identity as much as possible. From all the participants a written informed consent was obtained before the data collection began; therefore, each participant was aware of the research objectives and purposes, their specific duties and their rights to be released from the study with no explanation needed.

In the current study, the validated Arabic versions of oral health-related quality of life (OHRQoL) instruments, such as the OHIP-14, WHOQOL-BREF, and OIDP, were employed.^5,8,17,20^ OHIP-14 scores range from 0–56, with higher scores indicating poorer OHRQoL. OIDP scores range from 0–100, where higher scores reflect greater oral impacts on daily performance, and WHOQOL-BREF scores are transformed to a 0–100 scale, with higher scores indicating better quality of life. To confirm the reliability of these instruments, a pilot study with a subset of the target population was done, demonstrating high internal consistency (Cronbach’s α: OHIP-14 = 0.93, OIDP = 0.91, WHOQOL-BREF = 0.89). The pilot participants were excluded from the final data analysis.

Data collection was conducted in three phases: baseline assessment, treatment phase, and one-month follow-up. At baseline, participants were assessed prior to initiation of treatment. They were subsequently reassessed during treatment, and finally again at one month following completion of all restorative procedures. At the baseline visit, participants underwent a clinical examination including caries assessment via the Decayed, Missing, and Filled Teeth (DMFT) index and based on this score were divided into 4 caries severity groups: group A: highest caries severity (highest DMFT scores); group B: moderate caries severity; group C: low caries severity; group D: very low caries severity (lowest DMFT scores). In addition, sociodemographic questionnaires alongside the three OHRQoL instruments were completed.

During the treatment phase, standard restorative treatment was provided to address the oral health sequelae of untreated dental caries, including transient dental sensitivity or pain, food impaction, and functional limitations. Treatment interventions included direct restorations using composite resin and resin-modified glass-ionomer cement. No extractions or endodontic treatments were included in this study. All procedures were performed by trained dental clinicians (faculty members and supervised interns) under standardized clinical protocols. The restorative materials were selected according to the clinical indications, and the individual patient’s needs as determined by the treating clinicians. The provided restorative dental treatment details such as restoration type, number of surfaces restored, use of anaesthesia, and complications were documented in the patient’s record.

The follow-up visit was held approximately one month after treatment. At that point, the participants completed the three Arabic versions of OHRQoL instruments. Furthermore, during this follow-up visit, clinical re-evaluation was performed to assess the conditions of the restorations and to document any interim events such as postoperative pain or re-treatment needs. To ensure the quality of data, it was collected by trained and calibrated examiners using standardized procedures to ensure consistency and minimize measurement bias.

A distinct aspect of oral health was measured by each OHRQoL instrument. The frequency of negative impacts of oral conditions across seven domains was evaluated by the OHIP-14. The overall quality of life across psychological, physical, environmental and social domains was measured by WHOQOL-BREF.

SPSS version 31 (IBM; Armonk, NY, USA) was utilized to statistically analyze the collected data. Data cleaning techniques were applied to determine if there were any missing values or erroneous entries made into the data collection tool. Summary statistics (means, standard deviations, frequency, percent) were generated to describe the participants’ characteristics and to provide summary scores for the OHRQoL tools. Discriminant validity was evaluated through the use of independent t-test procedures that examined the differences in OHRQoL tool scores from participants who reported good versus poor self-perceived oral health and general health status. Paired t-test procedures were employed to compare the OHRQoL tool scores prior to treatment with those obtained after treatment. In addition to t-test comparisons, effect size (Cohen’s d) was calculated to determine the magnitude of change. Effect size interpretations included small (0.2 ≤ ES < 0.5), moderate (0.5 ≤ ES < 0.8), and large (ES ≥ 0.8). Utilizing both t-test procedures and effect size estimates added to the clarity and validity of the results

Finally, a stepwise multiple linear regression analysis was performed to identify predictors of instrument responsiveness. With variable entry and removal criteria set at a significance level of p = 0.05, the analysis controlled for pre-treatment scores.

## RESULTS

Of a total of 279 participants initially recruited, 269 completed the one-month follow-up, yielding a response rate of 96.4%. Ten participants were lost to follow-up due to non-attendance. Of these 269 patients, 102 were female (37.9%) and 167 were male (62.1%). The majority were above 19 years of age (72.9%), were enrolled in high school (56.9%), and had a low economic status (61.7%) (Table 1).

**Table 1:** General characteristics of the participants (n = 269)

Variable	N (%)
**Sex** Female Male	102 (37.9) 167 (62.1)
**Age**	
16-19	73 (27.1)
>19	196 (72.9)
**Education**	
Jr High School	60 (22.3)
High School	153 (56.9)
College: Bachelor	51 (19.0)
College: Master and above	5 (1.9)
**Economic status**	
Sufficient	30 (11.2)
Barely sufficient	73 (27.1)
Insufficient	166 (61.7)


Regarding oral health behaviors and clinical characteristics, the majority of the participants reported brushing their teeth once daily (75.5%), while 18.2% used dental floss, and only 2.2% used oral rinses. Additionally, 65.1% replaced their toothbrushes after more than six months, most did not have crowded incisors (78.4%), and 42.0% were classified under group B caries severity (Table 2).

**Table 2:** Oral examination, oral health behavior and treatment status distribution

Variable	N (%)
**Brushing frequency**	
Not at all	7 (2.6)
Once daily	203 (75.5)
Twice daily	59 (21.9)
**Use of dental floss**	
No	220 (81.8)
Yes	49 (18.2)
**Use of oral rinse**	
No	263 (97.8)
Yes	6 (2.2)
**Toothbrush replacement intervals**	
Within 3 months	36 (13.4)
4-6 months	58 (21.6)
Over 6 months	175 (65.1)
**Crowded incisors**	
No	211 (78.4)
Yes	58 (21.6)
**Caries severity group**	
Group A	84 (31.2)
Group B	113 (42.0)
Group C	19 (7.1)
Group D	53 (19.7)
Group A: highest; group B: moderate; group C: low; group D: very low.	

The participants reporting good oral health demonstrated statistically significantly higher mean OHIP-14 scores compared to those reporting neither poor nor good oral health (7.42 vs 5.20; p = 0.002). The discriminatory ability of all the measurements was calculated for perceived oral and general health conditions among the study participants. The mean OIDP score was statistically significantly higher among those participants reporting good oral health (0.31 vs 0.17; p < 0.001). The mean WHOQOL-BREF score was also statistically significantly higher among those with good oral health compared with those reporting neither poor nor good oral health (43.69 vs 41.27; p < 0.001). In addition, the WHOQOL-BREF scores were statistically significantly higher among those participants who reported good general health compared with those reporting average general health (43.45 vs 42.06; p = 0.004) (Table 3).

**Table 3:** The discriminatory ability for all measurements of perceived oral and general health conditions

Variables		OHIP		OIDP		WHOQOL-BREF	
	n (%)	Mean (SD)	p-value	Mean (SD)	p-value	Mean (SD)	p-value
Perceived oral health conditions			0.002		0.011		< 0.001
Neither poor nor good	74 (27.5)	5.20 (4.84)		0.17 (0.34)		41.27 (3.37)	
Good	195 (72.5)	7.42 (5.62)		0.31 (0.48)		43.69 (4.59)	
Perceived general health conditions			0.199		0.330		0.004
Neither poor nor good	82 (30.5)	6.41 (4.70)		0.29 (0.48)		42.06 (3.52)	
Good	187 (69.5)	6.98 (5.82)		0.26 (0.44)		43.45 (4.70)	


Upon completion of caries treatment, statistically significant improvements were observed for all OHRQoL measures. The mean score for the OIDP instrument demonstrated a substantial reduction from 7.17 to 0.27 (p < 0.001). Similarly, the mean score for OHIP-14 decreased from 20.64 to 6.81 (p < 0.001), with all its subdomains also showing a statistically significant decline post-treatment (p < 0.001). In contrast, the mean WHOQOL-BREF scores showed a statistically significant increase from 32.53 to 43.03 post-treatment (p < 0.001); the same pattern reflected across all its subdomains (p < 0.001) (Table 4).

**Table 4:** Responsiveness to dental caries treatment for all the measurements assessed in 269 subjects

	Pre-treatment	Post-treatment	Observed effect	Paired t-test	Effect size
Domains	Mean (SD)	Mean (SD)	Mean (SD)	p-value	
OIDP score	7.17 (3.82)	0.27 (0.45)	6.91 (3.59)	< 0.001	1.93
OHIP score	20.64 (10.23)	6.81 (5.50)	13.83 (5.26)	< 0.001	2.63
Function limitation	3.60 (1.77)	1.81 (1.43)	1.79 (0.59)	< 0.001	3.02
Physical limitation	4.18 (1.84)	1.82 (1.39)	2.36 (0.85)	< 0.001	2.79
Psychological discomfort	3.97 (2.06)	0.99 (1.00)	2.97 (1.25)	< 0.001	2.38
Physical disability	4.16 (2.11)	1.02 (0.95)	3.14 (1.33)	< 0.001	2.35
Psychological disability	1.58 (1.57)	0.40 (0.64)	1.18 (1.19)	< 0.001	0.99
Social disability	2.08 (1.53)	0.54 (0.80)	1.55 (1.07)	< 0.001	1.45
Handicap	1.09 (1.02)	0.24 (0.44)	0.85 (0.75)	< 0.001	1.14
WHOQOL-BREF score	32.53 (4.22)	43.03 (4.42)	10.50 (1.09)	< 0.001	9.64
Physical	6.93 (1.17)	8.96 (1.09)	2.03 (0.57)	< 0.001	3.56
Psychological	7.96 (1.21)	10.07 (1.33)	2.11 (0.79)	< 0.001	2.66
Social	7.94 (2.14)	11.90 (2.20)	3.96 (0.30)	< 0.001	13.37
Environmental	9.70 (0.95)	12.10 (0.95)	2.40 (0.36)	< 0.001	6.58


A multivariate linear regression analysis was conducted to adjust baseline scores, in order to identify factors associated with post-treatment OHRQoL changes. The severity of baseline dental caries (groups A and B) was found to be a statistically significant negative predictor of post-treatment OHIP-14 scores (p< 0.001 for both groups), indicating that participants having more severe initial caries experienced greater improvement on this measure. On the other hand, the caries status of group B was a statistically significant positive predictor of post-treatment WHOQOL-BREF scores (p = 0.006), suggesting an enhanced overall quality of life following treatment within this specific severity subgroup (Table 5).

**Table 5:** Association of all the measurements with the responsiveness after dental caries treatment

Variables	OHIP		OIDP		WHOQOL-BREF	
	β-value (95% CI)	p-value	β-value (95% CI)	p-value	β-value (95% CI)	p-value
**Sex** Female Male	1 0.31 (-1.05, 1.67)	0.654	1 0.11 (-0.01, 0.22)	0.058	1 -0.41 (-1.51, 0.68)	0.457
**Caries status** Group A Group B Group C Group D	-7.27 (-8.86, -5.68) -6.71 (-8.22, -5.20) -0.35 (-2.77, 2.08) 1	< 0.001 < 0.001 0.779	0.08 (-0.07, 0.24) 0.12 (-0.03, 0.27) 0.16 (-0.08, 0.40) 1	0.298 0.107 0.179	-1.34 (-2.85, 0.17) -2.00 (-3.44, -0.57) -0.47 (-2.77, 1.83) 1	0.083 0.006 0.689
**R2**	0.308		0.026		0.033	


## DISCUSSION

This prospective study among Saudi adults aimed to evaluate the responsiveness of three validated OHRQoL instruments, i.e., OHIP-14, OIDP, and WHOQOL-BREF, to assess the changes following restorative dental treatment for dental caries. Additionally, by exploring sociodemographic and behavioral determinants of oral health outcomes, this study also offers insights into perceived impact of treatment on the patients’ oral and general health.

The current study exhibits several methodological strengths. First, the clinical examinations of the participants were undertaken by a well-trained specialist in oral diagnosis, thus enhancing the accuracy and validity of the clinical data. Second, three instruments were employed, assessing both oral and general health domains, thus facilitating a comprehensive assessment of treatment effects. These three instruments were found to be highly reliable for this population as indicated by high Cronbach’s alpha scores for each instrument (OHIP-14 = 0.93, OIDP = 0.91, WHOQOL-BREF = 0.89), supporting their internal consistency. In addition, the results of the present study support the results from previous studies showing that these instruments are very stable and reliable when evaluating the OHRQoL and HRQoL for adult patients in need of restorative dental care for caries.^7,32^

Caries is not limited to children only; it can also affect adults throughout their entire lives. To date, there have been very few studies on the responsiveness of the OHRQoL and HRQoL measurements after restorative caries treatment in adults, specifically in Saudi Arabia.^4,18^ The sample in the present study was composed of young adults (predominantly male) from an economically disadvantaged background. This demographic profile is consistent with earlier research from Saudi Arabia and other Middle Eastern regions, indicating that suboptimal oral hygiene and a higher prevalence of untreated caries are more common among young adults and lower socioeconomic groups.^26^ The financial constraints reported by over 60% of participants in the current study may explain the observed insufficient preventive practices, including brushing only once daily (75.5%), infrequent flossing (18.2%), and delayed toothbrush replacement (65.1% after more than six months). These patterns reflect those observed in national surveys conducted in Saudi Arabia, indicating that oral hygiene behaviors in Saudi Arabia remain below international standards despite increased and improved accessibility to dental care services.

Notably, gender did not emerge as a statistically significant predictor of post-treatment responsiveness across any of the instruments used in the current study. This finding contrasts with previous studies, which have generally reported a greater perceived impact on OHRQoL among females.^31,32^ The existing gender disparity in current research may indicate cultural or contextual influences within the Saudi population, thereby affecting health reporting behaviors.

In our study, all three quality-of-life instruments demonstrated statistically significant responsiveness following restorative treatment for caries. In OHIP-14 and OIDP scores, the substantial reductions reflect marked improvements across functional, physical, psychological, and social dimensions of oral health. Conversely, in WHOQOL-BREF scores, the statistically significant increase indicates an enhanced overall quality of life across all its domains. The clinical significance of these changes was further substantiated by large standardized effect sizes (Cohen’s d = 2.63 for OHIP-14, 1.93 for OIDP, and 9.64 for WHOQOL-BREF). The OHIP-14 exhibited the greatest sensitivity to change, aligning with previous research that identifies it as a highly responsive instrument for measuring treatment-related improvements in OHRQoL.^3,27^

The results of the present study are consistent with the findings of earlier studies by Locker et al^19^ and the WHOQOL Group,^31^ who documented statistically significant post-treatment gains in OHRQoL, especially within domains such as physical pain and psychological discomfort. All seven OHIP subdomains improved statistically significantly in the present study, with the most significant improvements observed in psychological discomfort, physical disability, and physical limitation. This finding verifies the established literature indicating that oral diseases and their treatment greatly affects daily functioning and psychosocial well-being.^7,10^ However, these findings differ from those those of previous research reporting greater quality-of-life improvements after more invasive procedures, such as extractions or surgical interventions, and suggest that even conservative restorative treatments can confer meaningful psychosocial and functional benefits.^8,21^

The findings of the current study revealed that the severity of dental disease was a key determinant of responsiveness of QoL instruments. The participants who presented with more severe caries at the baseline visit (groups A and B) exhibited the greatest improvement in post-treatment OHIP scores, supporting the concept that restorative dental treatment impact is most pronounced among individuals with a higher initial caries burden. This finding aligns with previous research, thus demonstrating a direct relationship between untreated caries and diminished OHRQoL.^4,8,12,22^

Our findings underscore the importance of carefully selecting OHRQoL and HRQoL instruments, with careful consideration given to the study population, severity of disease, and treatment modality. Moreover, the implementation of standardized treatment protocols and the calibration of examiners greatly enhanced the reliability of the study’s results.

However, this study has several limitations that should be acknowledged. First, the relatively short follow-up period of one month may be insufficient to evaluate the long-term effects of treatment or the potential recurrence of the condition. Second, although validated quality-of-life instruments were used, the reliance on self-reported data introduces the possibility of response bias. The exclusion of individuals with systemic diseases or complex dental conditions may limit the generalizability of the findings, as the results may be more applicable to the relatively healthy population. This study relied on self-reported measures, which may be subject to response bias. Additionally, potential confounding variables such as dietary habits, access to care, and psychological factors were not controlled. Finally, the findings should be interpreted with caution, as the sample was clinic-based from one region of Saudi Arabia and may not be fully representative of the broader Saudi population.

Based on the results of the current study, it is recommended that future research should focus on studying the long-term effects of restorative dental treatment on the oral health-related quality-of-life (OHRQoL) and health-related quality-of-life (HRQoL) among diverse populations/cultural settings in the broader Middle East. Increasing the geographic range of studies will increase their generalizability and will provide data to develop regionally-specific interventions.

Furthermore, integrating evidence-based preventive strategies into routine restorative care is essential to address underlying behavioral risk factors, thereby maximizing the overall efficacy of dental treatment and improving patient-centered outcomes.

## CONCLUSION

The findings of this study confirm the positive impact of restorative treatment for caries on both the Oral Health-Related Quality of Life (OHRQoL) and general well-being in the adult Saudi population. The statistically significant and consistent post-treatment improvements across all the functional, psychological, and social domains of the OHIP-14, OIDP, and WHOQOL-BREF instruments demonstrate that the benefits of dental treatment extend beyond clinical outcomes and contribute substantially to patients’ overall quality of life and well-being. Therefore, these findings emphasize the value of integrating Patient-Reported Outcome Measures (PROMs) into standard dental practice. Hence, adopting this dual-focus model – combining clinical and psychosocial outcomes – is essential for advancing a holistic, patient-centered approach to oral healthcare.

## ACKNOWLEDGEMENT

This work was financially supported by the Deanship of Graduate Studies and Scientific Research at Jouf University under grant No. (DGSSR-2024-01-01122).
